# Combining attentional bias modification with dorsolateral prefrontal rTMS does not attenuate maladaptive attentional processing

**DOI:** 10.1038/s41598-018-37308-w

**Published:** 2019-02-04

**Authors:** Leonore Bovy, Martin Möbius, Martin Dresler, Guillén Fernández, Alan Sanfey, Eni Becker, Indira Tendolkar

**Affiliations:** 1 Donders Institute for Brain, Cognition and Behaviour, Radboud University Medical Center, Kapittelweg 29, 6525 EN Nijmegen, The Netherlands; 2 Behavioural Science Institute, Radboud University Nijmegen, 6500 HE Nijmegen, The Netherlands

## Abstract

High frequency repetitive Transcranial Magnetic Stimulation (rTMS) over the left dorsolateral prefrontal cortex (DLPFC) has been shown to reduce depressive symptoms and improve cognitive biases such as attentional bias. One promising technique that may complement rTMS treatment is attentional bias modification (ABM) training, given the similarity in modulating attentional bias and affecting neuronal activity. We tested whether the combination of rTMS treatment and ABM training in a single session would attenuate maladaptive attentional processing and improve mood in participants with subclinical depressive symptoms. To this end, 122 healthy participants were randomly assigned to one of four groups, receiving either a single rTMS treatment, a single ABM treatment, a combination of rTMS and ABM or a sham treatment. Of these 122 participants, 72 showed a heightened BDI-II score (between 9 and 25) and were included in our main analyses. In our subclinical (≥9 and ≤25 BDI-II) sample, a single combination treatment of rTMS and ABM training induced no significant changes in attentional bias, attentional control or mood, nor did rTMS alone affect attentional bias systematically. We discuss these null findings in light of the task specifics and relate them to the ongoing discussion on ABM training in depression.

## Introduction

The lifetime prevalence of major depressive disorder (MDD) is the highest among all mental disorders^1^. A range of different treatment options are available, with pharmacotherapy and cognitive behavioral therapy being the most frequently applied forms of treatment^2^. Unfortunately, a considerable number of patients do not benefit from these treatments or remain suffering from recurrent episodes after initially successful therapy^3,4^. After several recurrent depressive episodes, patients can be offered electroconvulsive therapy (ECT), a form of treatment that is accompanied by severe side effects such as anterograde and retrograde memory impairments^5^. Therefore, less invasive forms of therapy are urgently required for otherwise treatment-resistant patients^6^.

One promising alternative treatment option is repetitive transcranial magnetic stimulation (rTMS) therapy, which has repeatedly been shown to reduce depressive symptoms^7,8^ (for meta-analyses see ref.^9,10^; for consensus review see ref.^11^). TMS is a non-invasive technique used to stimulate local regions of the cortex. Repetitive TMS (rTMS), in comparison to single pulse stimulation, involves a train of magnetic pulses at a specific frequency, eliciting longer-lasting effects than the stimulation itself^12^. Depending on the frequency with which a magnetic pulse is emitted, neuronal stimulation can selectively activate or inhibit the cortical area under the coil^13^. rTMS seems to be a promising tool in treating depression as it is considered safe^8^, leads to relatively few and mild side effects, and is low in cost^14^. The left dorsolateral prefontal cortex (DLPFC) is a common stimulation site for rTMS treatment, as functional neuroimaging studies have shown a decrease in regional cerebral blood flow in this region in depressed patients^15,16^. This hypoactive state has been observed during depressive episodes as well as after remission^17^. Furthermore, rTMS stimulation over the left DLPFC, in addition to enhancing activation in the prefrontal cortex, also indirectly activates several subcortical structures related to the pathophysiology of depression, namely the anterior cingulate gyrus, putamen, hippocampus, and thalamus^18^.

Several meta-analyses have demonstrated statistically superior effects of rTMS treatment as an antidepressant tool as compared to control treatments^9,10,14,19,20^ (but see ref.^21^), however, the effect sizes are moderate^22,23^ and smaller than existing ECT treatments^24^. In addition, the durability of the anti-depressant effect after acute rTMS treatment appears small and decreases over time^25^. It has been shown that combining the effect of antidepressant medication and rTMS seems to enhance the overall antidepressant effect^26^, however, some antidepressant medications come with unwanted side effects. In line with these latter attempts though, it is therefore of particular interest to investigate the possibility of amplifying the beneficial effects of rTMS by combining it with psychological interventions.

Cognitive changes are a core feature of depression, including impairments in attention, memory and executive function^27^. In line with cognitive models of depression^28,29^, cognitive biases, characterized by a preferential processing of emotionally negative over positive information, have been shown to play a causal role in the development and maintenance of depression (for review see ref.^30,31^).

One technique that is very promising in complementing rTMS treatment is attentional bias modification (ABM) training, a computerized training method designed to alter cognitive biases. The most frequently used task to measure and modify attentional bias towards negative stimuli is the dot-probe task^32^. During this task, participants have to respond as quickly as possible to a dot presented behind one of two pictures (one neutral/positive, one negative). By increasing the frequency of the dot appearing behind the neutral/positive stimuli, participants’ attention can be selectively trained towards neutral/positive stimuli and away from negative valenced stimuli^33,34^. This form of ABM training has shown some success in modifying attentional bias and in turn in reducing emotional vulnerability in response to stressful situations even after a single session (for review see ref.^35,36^).

ABM might be well-suited to complement rTMS, as both techniques seem to overlap in the neural anatomy affected. ABM training increases neural activity in the left PFC and rostral ACC^37^, with greater activity when participants had to exert most cognitive control, that is, during task conditions where the direction of participants’ attention was contrary to the direction they were trained to attend to. In addition, ABM training is thought to increase top-down control, which could bolster control over attentional allocation^38^. However, these results are still unclear in depressive patients, as to date most results are based on anxiety studies^35,39^. Nonetheless some studies show that ABM can directly reduce depressive symptomatology in mild to moderately depressed participants after several training sessions^40–42^, but also see e.g. ref.^43^ for a failure to replicate). In addition, a computerized ABM training, when proven effective, is an easily implementable training as add-on therapy to current depression therapies. Repetitive TMS in turn has also shown to affect attentional processing, for example it has been shown that a single session of DLPFC rTMS can affect (non-emotional) attentional processing of both healthy and depressed patients^44–47^. Replicating these findings, DLPFC stimulation in a sample of healthy women has been demonstrated to reduce attentional engagement towards angry faces^48^, associated with increased activity within the right DLPFC, dACC, right superior parietal gyrus and left orbitofrontal cortex. This effect of rTMS over the DLPFC on attentional bias has been linked to prefrontally mediated processing of attentional processes^49^, for instance heightened attention towards negative stimuli, e.g. sad or angry faces^42^.

Taken together, these studies show that ABM and rTMS have comparable effects in both affecting the neuronal activity of the DLPFC and also modulating attentional biases for emotional information. ABM might thus be a promising and practical tool capable of maximizing the effects of rTMS and thus amplifying beneficial therapeutic effects in the treatment of depression. Furthermore, as prolonged high frequency stimulation of the left DLPFC increases the excitability of this brain region^23,50^, subsequent ABM treatment might make use of this increased cerebral activity, resulting not merely in the accumulation, but in the enhancement of the effects.

**Figure 1:**
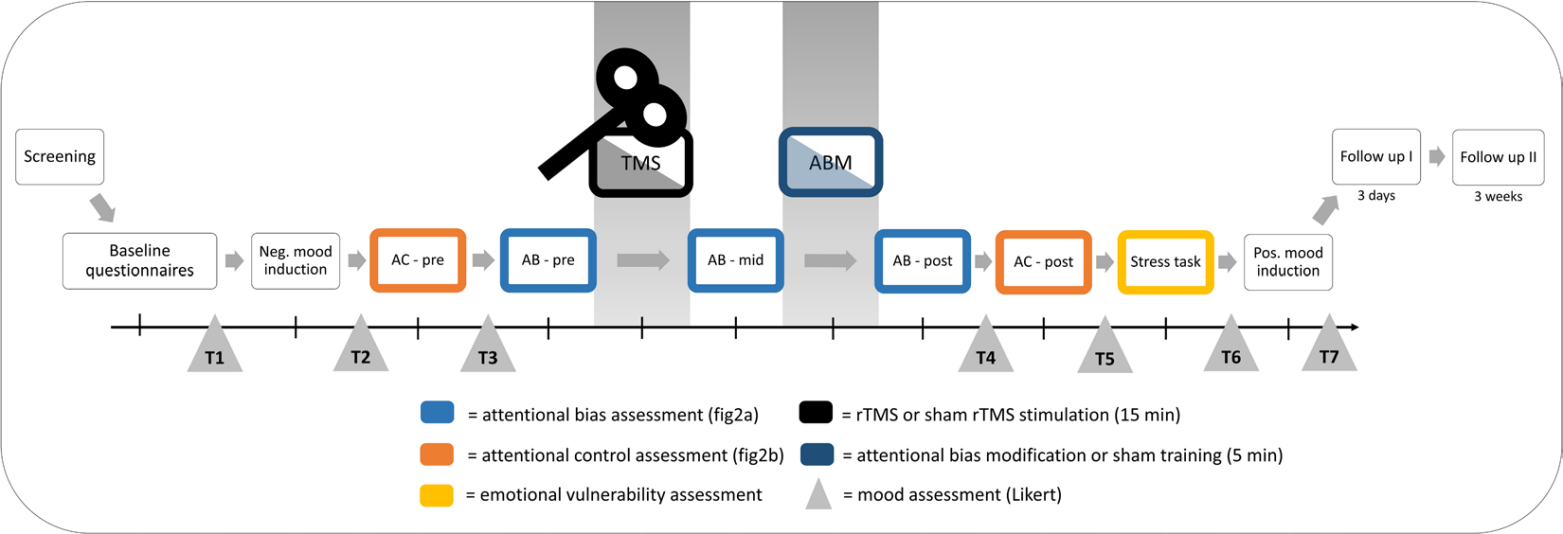
Task procedure and timeline. An online screening took place around two weeks before actual participation. Suitable participants were then invited for a lab session. After providing informed consent, participants were first prepared for the repetitive transcranial magnetic stimulation (rTMS) treatment and resting motor threshold (MT) was determined (not displayed here). Participants were assigned to one of 4 groups where they would either receive (1) an active rTMS with sham attentional bias modification (ABM) treatment, (2) a sham rTMS with active ABM treatment, (3) a combination of active rTMS and active ABM treatment, or (4) a sham rTMS and sham ABM treatment. Participants filled in several baseline questionnaires (10 minutes). Negative mood was induced through short movie clips (20 minutes). Then, the pre-assessment of the attentional control (AC - pre) task was performed (10 minutes) as well as of the attentional bias (AB- pre) task (5 minutes). Between the rTMS and ABM treatments, a second (mid-)attentional bias assessment (AB – mid) was performed (5 minutes), and a third (post-)assessment (AB – post) immediately after both treatments (5 minutes). Next, the post-assessment of the attentional control (AC – post) task (5 minutes) followed by a stress task (5 minutes) were performed. Lastly, a positive mood induction movie (5 minutes) was presented. Throughout the testing session, mood state was assessed by Likert scales (see grey triangles), at baseline (T1), after the negative mood induction (T2), before the pre-assessment of attentional bias (T3), after the post- attentional bias assessment (T4), after the attentional control post-assessment (T5), after a stress-inducing task (T6) and after the final positive mood induction (T7). Three days as well as three weeks after the laboratory session, follow-up measurements (BDI-II, STAI-T, PANAS) were administered. *Note: ms* = milliseconds, *AC* = attentional control, *AB* = attentional bias, *pos*. = positive, *neg*. = negative, *TMS* = transcranial magnetic stimulation, *ABM* = attentional bias modification. *BDI-II* = Becks depression inventory*; STAI-T* = Spielberger trait anxiety inventory*; PA* = positive affect from the Positive and Negative Affect Scale (PANAS)*; NA* = negative affect from the PANAS.

Initial evidence for these proposed synergistic effects comes from a study applying transcranial direct current stimulation (tDCS)^51^. During tDCS, a weak electric current is applied to the cortex by electrodes attached to the scalp, with the aim of altering the neural excitability of the cortical areas under the electrodes (for review see ref.^52^). In one study^51^, the authors combined tDCS with an ABM training in a sample of healthy individuals, showing that participants who received the tDCS stimulation to enhance activity of the left DLPFC had improved learning of the ABM training contingencies, as compared to participants who received sham-stimulation. In a subsequent study^53^, the authors demonstrated that such a change in attentional bias in healthy controls mediated the stress attenuating effects of the tDCS stimulation. However, to the best of our knowledge, these synergistic effects of ABM and neuromodulation have not been explored by implementing rTMS in a sample of vulnerable subjects. Indeed, before testing patients suffering from MDD in a fully controlled clinical trial, it is advisable to investigate possible effects in a single session approach applied to a non-clinical population, where a proper approximation of a depressive sample can be achieved by using an analogue depressed sample, that is, in individuals showing a heightened depression scores^54^. The aim of the current project therefore was to test the synergistic effects of adding ABM training to rTMS treatment in a randomized, sham-controlled manner, using a single session in an, otherwise healthy, sample with heightened depression scores, in line with ref.^40^. Sad mood was induced in all participants in order to activate latent depressogenic cognitive schemas^55^, thereby imitating an analogue depression. We hypothesized that combining both treatments would amplify beneficial effects by (1) reducing attentional bias as assessed by a computerized dot-probe task, (2) increasing attentional control as measured by a modified Stroop task and (3) decreasing negative mood as determined by several mood questionnaires (Likert, PANAS). In addition, we explored the effect of both treatments on emotional vulnerability through a stress-inducing incidental memory task. To this end, we chose a randomized controlled design where participants were assigned to one of 4 groups where they would either receive A) an active rTMS with sham ABM treatment (rTMS group), B) a sham rTMS with active ABM treatment (ABM group), C) a combination of active rTMS and ABM treatment (combination), or D) a sham rTMS and sham ABM treatment (control). Attentional bias was measured over three time points, namely before (pre-assessment), between (mid-assessment) and after (post-assessment) the rTMS and/or ABM treatments, to ensure distinguishability of main and interaction effects of the treatments. Attentional control was measured on two time points, namely before (pre-assessment) and after (post-assessment) the two treatments. Mood questionnaires were administered at the start and during the lab session as well as 3 days and 3 weeks later via online surveys (see Fig. 1 for a full procedure overview).

## Results

### Data reduction, outliers and group characteristics

One hundred and twenty-two participants were invited to the lab based on their screening BDI-II scores (≥9). Baseline BDI-II scores were again measured in the lab prior to testing, where 72 participants still matched the inclusion criteria of a BDI-II baseline score between 9 and 25. All subsequent results describe the sample after BDI-II score restriction, where only participants with a BDI-II baseline score between 9 and 25 are included, unless otherwise specified.

In addition, the sample sizes of the different measurements differed slightly because of missing data due either to technical difficulties or to removal of extreme individual median reaction time responses (i.e., scores more than 1.5 interquartile ranges below the first or above the third quartile) that affected normality. Specific sample sizes for all different analyses are reported in the Supplementary Materials (see S-Table 1).

Individuals in the 4 groups did not differ significantly on the demographic variables or baseline mood/trait characteristics indicating that potential findings were not confounded by basic differences between groups (see Tables 1 and 2).

**Table 1:** Group differences on demographic variables, mean and standard deviation are reported on restricted sample.

	rTMS (*N* = 21)	ABM (*N* = 18)	Combination (*N* = 18)	Control (*N* = 15)	
Age	21.48 (2.64)	20.67 (2.57)	21.17 (2.04)	22.36 (3.52)	*F(3*, *68)* = 1.97, *p* = 0.13
Gender					χ^2^*(3)* = 3.75, *p* = 0.29
Male	6	16	12	13	
Female	15	2	6	2	
Nationality					χ^2^*(3)* = 2.52, *p* = 0.47
Dutch	15	15	14	9	
German	6	3	4	6	

*Note: rTMS* = repetitive transcranial magnetic stimulation; *ABM* = attentional bias modification.

**Table 2:** Group differences on baseline questionnaires, mean and standard deviation are reported on restricted sample.

	rTMS (*N* = 21)	ABM (*N* = 18)	Combination (*N* = 18)	Control (*N* = 15)	
BDI-II	14.8 (5.33)	13.28 (4.23)	11.78 (2.62)	13 (3.55)	*F*(*3*, *68)* = *1*.*77*, *p* = *0*.*16*
STAI-T	49.1 (8.19)	47.67 (7.37)	49.11 (7.17)	50.73 (5.51)	*F*(*3*, *68)* = *0*.*49*, *p* = *0*.*69*
NA	22.81 (7.07)	23.4 (5.16)	24.22 (4.91)	24.27 (7.31)	*F*(*3*, *68)* = *0*.*24*, *p* = *0*.*87*
PA	28.09 (5.84)	29.06 (5.3)	31.83 (6.71)	29.93 (3.88)	*F*(*3*, *68)* = *1*.*07*, *p* = *0*.*37*
Likert	5.72 (1.39)	6.02 (1.16)	5.53 (1.29)	5.72 (1.39)	*F*(*3*, *68)* = *0*.*41*, *p* = *0*.*74*

*Note: rTMS* = repetitive transcranial magnetic stimulation; *ABM* = attentional bias modification; *BDI-II* = Becks depression inventory*; STAI-T* = Spielberger trait anxiety inventory*; PA* = positive affect from the Positive and Negative Affect Scale (PANAS)*; NA* = negative affect from the PANAS; *Likert* = mood assessment on 10-point psychometric rating scale.

### Effects on attentional bias

A 4 (group: rTMS, ABM, combination, control) × 2 (time: pre-assessment, post-assessment) mixed ANOVA on attentional bias was performed, revealing neither significant differences for time (*F*(1, 68) = 0.25, *p* = 0.618, η²_G_ = 0.002), nor between the groups (*F*(3, 68) = 1.08, *p* = 0.362, η²_G_ = 0.025). Moreover, we found no significant interaction effect for time and groups (*F*(3, 68) = 0.564, *p* = 0.641, η²_G_ = 0.012).

A JZS Bayes factor ANOVA^56^ with default prior scales was performed to estimate the likelihood of the interaction effect. This resulted in a BF_10_ of 0.005 (BF_01_ = 200), thereby providing extreme evidence for the absence of an effect^57^. The effect of time and group alone resulted in a BF_10_ of 0.209 (BF_01_ = 4.78) and 0.158 (BF_01_ = 6.33) respectively. See Supplementary Materials for a table on prior distributions (S-Table 2).

Since we measured attentional bias after the rTMS protocol and before the attentional bias training as a mid-assessment (see Fig. 1, light blue boxes), we decided to explore the isolated effect of rTMS on attentional bias with a larger sample, by pooling the two groups containing active TMS (with and without ABM) and sham TMS (with and without ABM). This lead to two larger groups of rTMS and sham treatment, with a sample size of rTMS (*n* = 37) and sham (*n* = 28). Again, a mixed ANOVA was performed to assess the effect of time (pre-assessment, mid-assessment) and group (active rTMS, sham rTMS) on attentional bias. The ANOVA revealed no significant differences for time (*F*(1, 70) = 0.674, *p* = 0.414, η2_G_ = 0.004), no significant differences between the groups (*F*(1, 70) = 0.352, *p* = 0.555, η2_G_ = 0.003), as well as no significant interaction effect for time and group (*F*(1, 70) = 0.324, *p* = 0.571, η2_G_ = 0.002) on attentional bias.

Again, a Bayes factor was calculated with default prior scales, which revealed a BF_10_ of 0.019 (BF_01_ = 52.63) for the interaction effect between group and time, thus providing very strong evidence for the null hypotheses of no effect. The effect of time and group alone resulted in a BF_10_ of 0.27 (BF_01_ = 3.7) and a BF_10_ of 0.251(BF_01_ = 3.98) respectively.

Note however that due to restricting the BDI-II scores and performing an ANOVA requiring a balanced design over time, our main analysis was performed on a smaller sample size (see S-Table 1).

We performed further exploratory analyses on the full sample of 122 subjects, which did not change the outcome of the analyses (see Supplementary Materials).

### Effects on attentional control

A modified Stroop task with arrows (including a written word “left” or “right” and displayed as symbols either pointing left or right) was used to assess individual levels of attentional control. By subtracting the reaction times of the congruent (word and symbol matched) from incongruent trials (word and symbol did not match), attentional control scores were calculated per participant per assessment. Attentional control was calculated at two time points: the pre-assessment time point before the rTMS and/or ABM treatments and the post-assessment time point after the treatments (see Fig. 1, orange boxes). A 4 (group: rTMS, ABM, combination, control) × 2 (time: pre-assessment, post-assessment) mixed ANOVA on attentional control revealed a significant main effect of time *F*(1, 62) = 13.236, *p* < . 001, η^2^_G_ = 0.08, indicating an overall decrease in difference scores over time (pre- assessment: *M* = 61.31, *SD* = 33.68, post- assessment: *M* = 43.73, *SD* = 29.93), presumably indicating a practice effect over time. There was no significant effect of group, *F*(3, 62) = 0.255, *p* = 0.857, η^2^_G_= 0.007, nor an interaction of group by time, *F*(3, 62) = 1.93, *p* = 0.134, η^2^_G_  = 0.03.

A Bayes factor was calculated with default prior scales, which revealed a BF_10_ of 3.03 (BF_01_ = 0.33) for the interaction effect between group and time, thus providing some anecdotal evidence for an effect in the data. This evidence for an interaction effect is however very small. Given the conflicting result from the previous frequentist statistics which resulted in a p-value of 0.134 with a small effect size, our alternative hypothesis of the presence of an interaction effect is not clearly confirmed. The data however provided very strong evidence for the effect of time, with a BF_10_ of 55.96 (BF_01_ = 0.018). Moderate evidence for the effect of group was found, with a BF_10_ of 5.16 (BF_01_ = 0.19), in favor for the alternative hypothesis.

Again, analyses were repeated on the full sample, yielding similar results (see Supplementary Materials for detailed results).

### Effects on mood, depression and anxiety scores

To examine the effects of the mood induction, a repeated measures ANOVA was conducted on self-report mood as measured by Likert scales before and after watching the mood inducing movies (T1 and T2 in Fig. 1 respectively) on the full sample (see Supplementary Materials for detailed results on restricted sample). The results yielded a significant main effect of time (*F*(1, 120) = 231.48, *p* < 0.001, η^2^_G_ = 0.38), where mood levels dropped (indicating an increase in negative mood) after watching the negative mood inducing movie, similarly for all groups. This analysis was repeated with mood scores assessed at T6 and T7 indicating elevated mood levels after watching the positive movie (*F*(1, 119) = 224.59, *p* < 0.001, η^2^_G_ = 0.29).

To test whether participants’ mood ratings changed after the rTMS and/or ABM manipulations, a 4 (group: rTMS, ABM, combination, control) × 2 (time: pre-manipulation, post-manipulation, T3 and T4 in Fig. 1 respectively) mixed ANOVA on Likert scales was performed. The ANOVA revealed no significant effects of group (*p* = 0.83) or interaction effects (*p* = 0.76). There was a significant effect of time (*F*(1, 117) = 73.57, *p* < 0.001, η2_G_ = 0.12), where all groups reported an increase in negative mood, possibly due to fatigue.

To test whether groups responded differently in their overall mood rating after a stressful task, another 4 (group: rTMS, ABM, combination, control) × 2 (Time: pre-stress task, post-stress task; T5 and T6 in Fig. 1 respectively) mixed ANOVA on Likert scales was performed. The ANOVA revealed no significant effects of group (*p* = 0.78) or interaction effects (*p* = 0.61). Again, there was a significant effect of time (*F*(1, 117) = 98.91, *p* < 0.001, η2_G_ = 0.116), where all groups reported an expected decrease in mood.

Mixed ANOVAs on BDI-II, STAI-T, PA and NA scores were performed, to test a differential change between groups on depression, anxiety and mood scores over time on both the full and the restricted sample. The ANOVAs revealed no significant differences over time, between groups nor interaction effects. For the restricted sample, a 4 (group: rTMS, ABM, combination, control) × 3 (time: baseline, 3 day follow up, 3 week follow up) mixed ANOVA on BDI-II scores was performed, which revealed a small main effect of time *F*(2, 104) = 3.54, *p* = 0.032, η^2^_G_ = 0.002, but no significant effect for group *F*(3, 52) = 1.38, *p* = 0.259, η^2^_G_ = 0.055, nor an interaction of both, *F*(6, 104) = 0.716, *p* = 0.638, η2_G_ = 0.011. Overall, BDI scores decrease over time, irrespective of treatment (baseline: *M* = 13.05, *SD* = 3.98, after 3 days: *M* = 12.38, *SD* = 4.83, after 3 weeks: *M* = 11.61, *SD* = 5.29). In addition, a 4 (group: rTMS, ABM, combination, control) × 3 (time: baseline, 3 day follow up, 3 week follow up) mixed ANOVA on STAI-T scores was performed, which revealed no significant main effect of time *F*(2, 110) = 0.70, *p* = 0.497, η^2^_G_ = 0.012, nor for group *F*(3, 55) = 0.706, *p* = 0.552, η^2^_G_ = 0.037, nor an interaction of both, *F*(6, 110) = 0.938, *p* = 0.471, η^2^_G_  = 0.048. Furthermore, another 4 (group: rTMS, ABM, combination, control) × 3 (time: baseline, 3 day follow up, 3 week follow up) mixed ANOVA on negative affect (NA) scores was performed, which revealed no significant main effect of time *F*(2, 110) = 0.93, *p* = 0.397, η^2^_G_ = 0.016, nor for group *F*(3, 55) = 0.176, *p* = 0.912, η^2^_G_ = 0.010, nor an interaction of both, *F*(6, 110) = 0.729, *p* = 0.627, η^2^_G_ = 0.038. Lastly, the 4 (group: rTMS, ABM, combination, control) × 3 (Time: baseline, 3 day follow up, 3 week follow up) mixed ANOVA on positive effect (PA) scores revealed no significant differences between the groups (*F*(3, 52) = 0.26, *p* = 0.854, η^2^_G_ = 0.015). There was however a small main effect of time (*F*(2, 104) = 3.262, *p* = 0.042, η^2^_G_ = 0.057) but no interaction effect (*F*(6, 104) = 0.74, *p* = 0.619, η2_G_ = 0.039) between time and group. PA values fluctuated over time as follows: baseline: *M* = 29.75, *SD* = 5.71, after 3 days: *M* = 28.8, *SD* = 5.22, after 3 weeks: *M* = 29.9, *SD* = 5.23 (see Supplementary Materials for detailed results for full sample).

## Discussion

The purpose of the present study was to investigate the possible synergistic effects of combining rTMS and ABM treatment on attentional bias, attentional control, and mood. Specifically, we aimed to replicate the beneficial effect of rTMS and ABM treatment separately on these measurements, and, more important, investigate whether combining both treatments results in stronger effects. In addition, the current study was performed on a clinically relevant sample of dysphoric students, where sad mood was successfully induced in order to activate latent depressogenic schemas^52^. However, our findings not support our predictions.

The combined effect of both treatments did not yield any significant differences as compared to the control conditions. Furthermore, Bayesian analyses provided strong support for the null hypothesis of no effect over our alternative hypothesis in our main analyses. In addition to the attentional bias, we explored the effects of ABM training and rTMS on attentional control, expecting an increase in flexibility in changing attention allocation after either or both manipulations. Again, no group differences were found, with all groups showing an increase in attentional control, suggesting a practice effect alone. Lastly, we observed no differences in emotional vulnerability between groups, as assessed by mood changes after the challenging and stressful task, nor any long-term effects of the manipulations on changes in depression or anxiety scores over time.

Even though several analyses were performed on a smaller subsample, analysis on the full sample resulted in similar findings, providing cumulative evidence that in the current study the rTMS intervention did not affect attentional processing. In addition, for the main effect of rTMS on attentional bias in particular, the rTMS groups were pooled and compared to the pooled sham-groups (independent of ABM treatment). With this sufficiently powered sample, no effect of rTMS on attentional bias could be observed. In combination with the Bayesian analyses, strong support was provided in favor of no effect on the difference between the groups on attentional bias. These results are in direct conflict with previous reports^58^, and suggest more thorough investigation and replication. Given that we did not find support for the effects of the isolated treatment techniques, it is conceivable that we were not able to detect any potential effect of the combination of both treatments either.

A possible reason for not detecting an effect of either or combined treatments could be the related to the dosage. This study employed a single session of rTMS, which might have been insufficient to induce a difference between groups on subtler processes such as attentional bias and control. Indeed, the effects of cumulative sessions of rTMS increase effectiveness of subsequent TMS-mediated behavioral processes^59^. Studies reporting on the influence of single rTMS sessions on cognitive performances are scarce. Previous results mainly report no differences in mood after a single session of high-frequency rTMS^46,54,60^, which is in line with our findings. However, given the few studies that did find an effect of single session rTMS on several cognitive performances, such as attentional control^46,60^, it remains to be proven, whether a higher dosage can result in larger effects. However, multiple ABM sessions are not necessarily more effective than single sessions^60,61^. A recent meta-analysis^39^ even indicates that a single ABM training is more effective in bias change as compared to multiple sessions. Therefore, our failure to replicate an effect of a single ABM session is not in line with previous works.

The dot-probe task was employed in the current studies for several reasons; first, it is the most frequent administered paradigm to alter attentional bias in anxiety and depression^35,36^. Second, it is a good fit with rTMS stimulation given the neurocognitive similarities^37,51^ and third, when proven effective, it is a convenient form of add-on therapy to current depression treatments or therapies.

A critical remark should however be made to the validity of the task. Even though several studies have succeeded in modifying a negative attentional bias in dysphoric individuals by using the dot-probe paradigm^40,41^, a range of studies have failed to replicate these effects^43,62^ and recent meta-analyses question the efficacy of ABM procedure for depression^35,39^. The dot-probe task has thereby been criticized for its low reliability in assessing attentional bias^63,64^. Importantly, the purely reaction-time based measurement might be insufficient to reliably assess for depression-relevant late disengagement from negative stimuli^65^ and might require additional eye-tracking recordings to measure attentional gaze direction^66^. This insight suggests that future studies make use of additional measurements other than only reaction times in endeavors exploring attentional biases.

Our study was employed on a clinically relevant sample of dysphoric students. Attentional bias to negative information has been found in dysphoric individuals^58,59^ as well as in depressed patients. We recommend future studies aiming at investigating such a combined intervention to rely on similar at-risk samples, which provide an approximation of a depressive sample, while at the same time minimizing the burden for patients.

Taken together, our study did not replicate effects of rTMS or ABM training on attentional bias or attentional control in a dysphoric sample. Furthermore, we could not confirm our hypothesis on the beneficial effect of combining these two techniques. The present study, in the context of recent null-findings, provides further indications that the dot-probe ABM training in its current form is not ready for large-scale dissemination as an antidepressant treatment, neither by itself nor in combination with rTMS treatment, as the evidence of a positive effect is still debatable. Also, the current study builds on the literature of null findings in rTMS treatment on attentional bias. These results are of great importance given the greater widespread use of rTMS treatment in depression related disorders. More studies on the specific cognitive effects of rTMS treatment are needed^67^, specifically in clinical settings. Given the enduring high prevalence of depression and relapse, the need for proper treatment is urgent.

## Methods

### Participants

One hundred and twenty-two Dutch-speaking participants (Age: *M* = 21.5, *SD* = 2.97) met the inclusion criteria and participated in return for course credit points or €25. Participants were semi-randomly assigned to 4 experimental groups, stratified by gender, receiving either: a single rTMS with sham ABM treatment (rTMS group, *n* = 32), a single ABM with sham rTMS treatment (ABM group, *n* = 30), a combination of rTMS and ABM treatment (combination, *n* = 30), or a sham rTMS and sham ABM treatment (control, *n* = 30; see Supplementary S-Fig. 1 for more details).

All participants were screened via online questionnaires prior to participation on several exclusion criteria regarding contraindication for TMS participation^68,69^, such as (family) history of epilepsy and neurosurgical interventions, prior head trauma, medication, having metal or magnetic objects in the body or being pregnant. Furthermore, participants were only included if they had a heightened score (between 9 and 25) on the Beck’s Depression Inventory (BDI-II^63^). These scores were chosen in line with previous ABM studies with dysphoric individuals^40,43^, in order to maximize the sensitivity of the BDI-II scale^64^. This study was conducted in accordance to the Declaration of Helsinki and approved by the local medical ethical committee (CMO Region Arnhem-Nijmegen, the Netherlands) and was registered at Netherlands Trial Register (NTR4637).

### Questionnaires

In order to measure individual differences in depressive symptoms, as well as for screening purposes, the Becks Depression Inventory (BDI-II) was used. Individual levels of trait anxiety were assessed with the Spielberger State-Trait Anxiety Inventory (STAI-T^62^). To assess general levels of affect the Positive Affect Negative Affect Schedule (PANAS^70^) was administered, assessing mood states of the past two weeks. To examine the time-course mood state and stress levels throughout the experiment, participants were asked to rate 6 statements on a 10-point Likert scale. These six statements separately assessed how tense, happy, nervous, relaxed, sad and content the participants were on a scale of 0–9. A total sum score represented overall mood, where questions were appropriately inversed such that a higher score reflects a better mood. See the Supplementary Materials for a more elaborate description of each questionnaire.

### Attentional bias task

Attentional bias was assessed at three time points (Fig. 1, blue boxes) and trained at one time point (Fig. 1, dark blue box) using an adapted version of the original computerized visual dot-probe task^33,65^. For this task, photos of 22 characters (11 female, 11 male) from the Radboud Faces Database were used, once displaying a happy and once a sad facial expression^66^. Pictures of 8 of these characters were presented during the assessment and the remaining 14 were presented during the training. In addition, 64 positive and negative pictures were selected from the Nencki Affective Picture System (NAPS^71^) representing different categories (e.g., humans, animals, scenarios). Of this selection, 24 pictures were presented during the assessment, while the remaining 40 pictures were presented during the training. For the assessment as well as the training, a positive picture was combined with a negative picture from the same data base.

**Figure 2:**
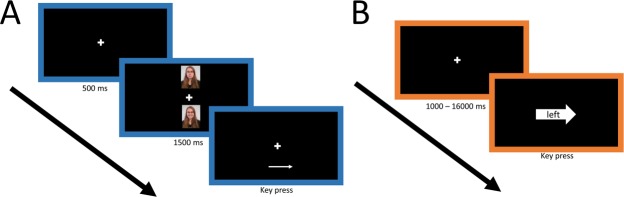
Attentional control and attentional bias tasks (**A)** Attentional bias (AB) task. A white fixation cross was presented in the center of a black screen. After 500 ms, a positive and a negative picture appeared above and below the fixation cross. After 1500 ms, both pictures disappeared and an arrow replaced one of the pictures, either pointing to the left or to the right. Participants had to respond to the direction of the arrow. The image depicts an example on a positive trial. (**B**) Attentional control (AC) task. A white fixation cross appeared in the center of a black screen. After a jittered interval between 1000 ms and 1600ms, an arrow pointing left or right, or a box replaced the fixation cross. The arrow or the box contained a word (i.e., *left* or *right*) that was either congruent or incongruent with the figure. Participants had to respond to the word by indicting the implied direction. The image depicts an example on an incongruent trial.

During each assessment, a white fixation cross was presented for 500 ms in the middle of the black computer screen. Then, two pictures appeared above and below the location of the fixation cross respectively, always a combination of a positive and a negative one. The above/below location of the positive and the negative picture was randomized across trials. After 1500 ms, both pictures disappeared and an arrow replaced one of those stimuli, either pointing to the left or to the right. Participants had to indicate the direction of the arrow as quickly as possible by pressing a designated button on the keyboard. Thereafter, the arrow disappeared and a new trial started. The direction of the arrow was counterbalanced across valences and position on the screen (i.e., above or below, see Fig. 2A).

During the training, these contingencies were changed such that the arrow replaced the positive pictures in about 85% of the trials. Participants in the sham training group received continued assessment, whereby the arrow appeared behind the positive picture in 50% of the trials. In addition, during the training, the presentation time of the pictorial stimuli was randomized between 500 ms and 1500 ms in order to prevent participants from predicting the timing of the appearance of the arrows, and in turn to keep their attention on the task. The length of the stimulus presentation was specifically chosen at a mean of 1000 ms, as longer stimulus durations have been shown to allow the participants enough time to optimally process the stimulus, aiming to optimally measure disengagement bias^40,72^.

Each assessment consisted of 72 trials, while we additionally presented 10 practice trials during the pre-assessment, and 2 practice trials during the second and third assessment. The training was presented after the second bias (mid-) assessment (see Fig. 1, dark blue box), consisting of 239 trials.

An attentional bias score was calculated for each assessment time point (pre-, mid- and post-assessment) separately, by subtracting the median reaction times on trials where the arrow replaced the positive picture from trials where the arrow replaced the negative picture. A positive score indicates a bias towards positive pictures, whereas a negative score implies a bias towards negative pictures. Reaction times that were faster than 200 ms were removed to eliminate fast guesses. In addition, latencies above or below 3 standard deviations of the individual mean were removed.

### Attentional control task

In order to measure individual differences in attentional control, a modified version of a Stroop task^73^ was administered. The task consisted of 90 trials. On every trial, a white fixation cross was displayed on the center of a black screen. After a jittered interval between 1000 ms and 1600 ms, an arrow either pointing to the left or to the right, or a box appeared at the central fixation cross in the middle of the screen. The arrow or the box contained the word “left” or “right” in it, to which the participant had to respond as quickly and accurately as possible by pressing the button corresponding to the direction the word was referring to (see Fig. 2B). The task consisted of three categories of trials: neutral, congruent, and incongruent. If the word matched the direction the arrow was pointing to, the trial was considered a “congruent” trial, whereas if the word and the direction of the arrow did not match, the trial was considered “incongruent”. If the word was presented in a box, without the presentation of a directional arrow, the trial was considered “neutral”.

A measure of attentional control was determined by calculating a difference score by subtracting reaction times of the incongruent trials from the reaction times on congruent trials, which indicated attentional control, with higher positive scores reflecting better control. Control was calculated for both assessments. Reaction times that were faster than 200 ms were removed to eliminate fast guesses. In addition, latencies above or below 3 standard deviations of the individual mean were removed.

### Stress-inducing Memory Task

In order to induce a heightened stress response, to assess emotional vulnerability, an emotional and challenging memory task was conducted (for details on the memory task, see Supplementary Materials).

### Mood induction

The negative mood induction procedure was adapted from ref.^74^. Two clips were selected from the movie ‘Sophie’s Choice’, together lasting about 20 minutes. Participants were instructed to place themselves into the position of the main actor to fully perceive the negative emotion. At the end of the experimental session, a positive mood induction was administered by presenting a segment from the movie ‘Happy Feet’, which has been demonstrated to induce positive affect^74^.

### rTMS procedure

Repetitive transcranial magnetic stimulation (rTMS) and sham treatment was administered using a MagPro-X100 stimulator (MagVenture, Farum, Denmark) and a C-B60 Butterfly (figure-of-eight) coil. F3 was the site of stimulation, which was marked in accordance to the international 10–20 system (American Electroencephalographic Society, 1994), as it had been recommended as an accurate procedure to identify the DLPFC without the use of neuro-navigation^75^. Stimulation intensity was set to 110% of the resting motor threshold (MT) of the right abductor pollicis brevis. Participants received 30 trains of 10 Hz pulses with a duration of 5 seconds and an inter-train interval of 25 seconds applied to the left DLPFC (50 pulses per train, 1500 pulses per session). The sham group received the same treatment, but had the coil tilted at a 45-degree angle, so that the participant was aware of the clicking of the pulses sent through the coil and received some scalp sensation, though the magnetic field was directed outside of the cortex.

### Procedure

For an overview of the experimental procedure see Fig. 1. After screening, participants fulfilling all selection criteria were invited to the laboratory session, where they were semi-randomly assigned to one of the four groups, stratified by gender. After providing informed consent, participants were first prepared for the rTMS treatment, which consisted of the resting MT determination and head registration using Localite (TMS-Navigator, Sankt Augustin, Germany), enabling us to easily navigate to F3 throughout the session. Subsequently, participants filled in a set of baseline questionnaires, consisting of the BDI-II, STAI-T and PANAS. Next, the negative mood induction was presented. Then, participants performed a baseline assessment (pre-assessment) of attentional control and attentional bias, which was followed by the (sham) rTMS stimulation. Immediately thereafter, a second attentional bias assessment was administered (mid-assessment), followed by the attentional bias modification training and the final assessment (post-assessment) of attentional bias. Then, the post-assessment of the attentional control task was performed followed by the challenging memory task. Before participants went home, the positive mood induction was presented. Throughout the testing session, mood state was assessed at baseline (T1), after the negative mood induction (T2), before the pre-assessment of attentional bias (T3), after the post-assessment of attentional bias (T4, about 30 min. after stimulation), after the post-assessment of attentional control (T5; about 40 min. after stimulation), after the stress-inducing memory task (T6; about 45 min. after stimulation) and after the final positive mood induction (T7; about 60 min. after stimulation). At the end of the 2.5 h testing session, participants were paid. Three days following the laboratory session, and again after three weeks, follow-up measurements were administered consisting of online questionnaires, including the BDI-II, the STAI-T and the PANAS.

### Data analysis

We analyzed the effect of the 4 groups (rTMS, ABM, combination and control) on attentional bias, attentional control and mood over several time points with mixed ANOVA designs, using the ezANOVA package^76^ in the R environment^77^. All post-hoc analysis were computed with the “HSD.test” function from the agricolae package^78^ to control for multiple comparisons by means of Tukey’s test. In addition, Bayes factors were calculated for the major results (attentional bias and attentional control) using JASP statistical software version 0.8.5^79^ to corroborate significant effects and quantify the support for the effects of interest. A BF_10_ < 1 provides weighted evidence in favor of the null hypothesis (H0), while BF_10_ > 1 supports weighted evidence in favor for the alternate hypothesis (H1). In turn, a BF_01_ contains the same information, as BF_01_ = 1/BF_10_^56^. For example, a BF_10_ of 10 suggests the alternative hypothesis is 10 times more probable than the null hypothesis, whereas a BF_10_ of 0.1 would suggest the null hypothesis is 10 times (1/0.1) more probable than the alternative hypothesis. BF_10_ values were interpreted as either anecdotal (1–3), moderate (3–10), strong (10–30), very strong (30–100) or extreme (>100) evidence for H1, whereas the same ranges for BF_01_ would provide evidence for H0^80^. In turn, BF_10_ or BF_01_ values below zero were interpreted as evidence for the null/ alternative hypothesis (respectively), in similar cutoff ranges. We ran JZS Bayes Factor repeated measures ANOVAs with default uniform prior scales as specified by the JASP software (r scale fixed effects = 0.5)^55^. The available datasets generated and/or analyzed during the current study are available from the corresponding author on reasonable request.

## Supplementary information

Supplementary materials
