# Supplementary material for: Combining attentional bias modification with dorsolateral prefrontal rTMS does not attenuate maladaptive attentional processing
Source: Sci Rep. 2019 Feb 4;9:1168. doi: 10.1038/s41598-018-37308-w (PMC6362221; doi:10.1038/s41598-018-37308-w)
Supplement: Supplementary file 1 — Supplementary materials [file 41598_2018_37308_MOESM1_ESM.pdf]

Supplementary materials

Title: Combining attentional bias modification with dorsolateral prefrontal rTMS does not attenuate maladaptive attentional processing

Authors: Leonore Bovy, Martin Möbius, Martin Dresler, Guillén Fernández, Alan Sanfey, Eni Becker, Indira Tendolkar

Methods

BDI-II. In order to measure individual differences in depressive symptoms as well as for screening purposes, the Becks Depression Inventory was used (BDI-II; Beck, Steer, & Brown, 1996). Participants had to indicate on 21 items, in how far these items describe themselves on a scale ranging from 0 to 3 resulting in a sum score between 0 and 63. The total score can further be divided into four ranges, describing the severity of depressive symptoms from minimal (0-13), mild (14-19), moderate (20-28) to serious depressive symptoms (29-63). All of these items describe the symptoms of major depressive disorder according to the DSM-IV (APA, 1994). The Dutch and German translation were used in this study and have shown to have good psychometric properties (Kühner, Bürger, Keller, & Hautzinger, 2007; Van der Does, 2002).

STAI-T. To assess individual levels of trait anxiety, the Spielberger State-Trait Anxiety Inventory was used (STAI-T; Spielberger, Gorsuch, Lushene, Vagg, & Jacobs, 1983). Participants had to indicate on 20 statements, how well they describe their general level of anxiety on a 4-point scale, ranging from 1 (almost never) to 4 (almost always). A total score is calculated ranging from 20 to 80, with higher scores representing higher trait anxiety. The Dutch and German translation were used in this study and have been shown to have good psychometric qualities (Laux, Glanzmann, Schaffner, & Spielberger, 1981; Van der Ploeg, Defares, & Spielberger,1980).

PANAS. The Positive Affect Negative Affect Scale (PANAS; Watson, Clark, & Tellegen, 1988) is a questionnaire of two 10 item mood scales in which is rated on a 5-point scale to which degree certain feelings such as ´hostile´ and ´proud´ were present the past two weeks. Items are divided in two categories, Positive Affect and Negative Affect. In each category scores can vary between 10 and 50, with higher scores representing higher levels of positive and negative affect respectively. The scales are found to be internally consistent and have a good convergent and discriminative correlation with lengthier measures of underlying mood factors (Crawford & Henry, 2004). The Dutch and German translation were used in this study (Krohne, Egloff, Kohlmann, & Tausch, 1996; Peeters, Ponds, & Vermeeren, 1996).

Screening questionnaires. All questionnaires were presented in the dominant language of the participants (i.e., Dutch or German). Before the experimental session, participants completed two screening questionnaires containing (1) transcranial brain stimulation related items (based on Rossi, et al., 2011) and (2) the Becks Depression Inventory (BDI-II) to assess levels of depression scores.

Stressful Memory Task

For the emotional memory task, 56 pictures (i.e., 28 positive, 28 negative) that were previously presented during the ABM training were used. A mirrored version of these pictures was shown 50% of the time (i.e., 14 positive, 14 negative), counterbalanced across faces and scenes. The remaining 28 pictures were presented in the same way as during the ABM task. A trial looked as follows: The participant was shown a white fixation cross on a black screen for 500 ms, which was replaced by a single picture. As soon as the participant indicated whether the presented picture was mirrored or not (i.e., by pressing a designated button on the keyboard) the picture disappeared and a new trial started. If the participant did not respond

within 1500ms or made an error, feedback (i.e., the word ‘incorrect’) was presented on the screen, accompanied by a loud noise asking the participant to react faster. Time pressure was implemented in order to induce stress.

Results

Attentional bias

Full sample. We explored if there was an effect of the full sample without restricting the BDI-II scores by means of a 4 (group: rTMS, ABM, combination, control) X 2 (time: pre-assessment, post-assessment) mixed ANOVA on attentional bias. The ANOVA revealed no significant differences for time ($F(1,118) = 1.354$, $p = .247$, $\eta^2 = .006$), no significant differences between the groups ($F(3,118) = 1.219$, $p = .306$, $\eta^2 = .015$), as well as no significant interaction effect for time and group ($F(3,118) = .907$, $p = .44$, $\eta^2 = .011$) on attentional memory bias. Again, we decided to test the isolated effect of rTMS on attentional bias with a larger sample, by pooling the two groups containing active TMS (with and without ABM) and sham TMS (with and without ABM). This lead to a sample size of active rTMS = 62 and sham rTMS = 60. Again, a mixed ANOVA was performed to assess the effect of time (pre-rTMS, post-rTMS) and group (Active rTMS, sham rTMS) on attentional bias. The ANOVA revealed no significant differences for time ($F(1,120) = .972$, $p = .326$, $\eta^2 = .000$), no significant differences between the groups ($F(1,120) = .023$, $p = .879$, $\eta^2 = .003$), as well as no significant interaction effect for time and group ($F(1,120) = .593$, $p = .443$, $\eta^2 = .002$) on attentional memory bias.

In addition, in further exploratory analysis, we looked into the bottom trials selectively, as previous studies have shown that bottom trials are more reliably assessing attentional bias[1] due to general eye gaze to top stimuli, regardless of emotional valence. Disengagement from the top stimuli is thus better measured in reaction times to the bottom stimuli.[2] In addition, as bottom trials are not affected by reading direction[2] heightened vigilance is suggested to be more reliably measured in bottom trials. Looking at only the bottom trials, we explored if there was an effect of the full sample with a 4 (group: rTMS, ABM, combination, control) X 2 (time: pre-assessment, post-assessment) mixed ANOVA on bias change was performed. The ANOVA revealed no significant differences for time ($F(1,118) = .596$, $p = .442$, $\eta^2 = .002$), no significant differences between the groups ($F(3,118) = .299$, $p = .826$, $\eta^2 = .004$). However, a significant interaction effect for time and group was found ($F(3,118) = 3.3$, $p = .023$, $\eta^2 = .037$) on attentional memory bias. Post-hoc analysis revealed a significant difference between the ABM ($M = -8.64$, $SD = 35.75$) and rTMS ($M = 18.79$, $SD = 28.26$) group at timepoint 2, $p = .012$, 95% CI [-50.31, -4.56] as well as a significant difference between the combination of treatments ($M = -4.02$, $SD = 33.09$) and rTMS groups, $p = .045$, 95% CI [-45.28, -0.334]. Notably, the difference between the control ($M = -1.83$, $SD = 37.27$) and the rTMS groups was in a similar direction, but not significant, $p = .089$, 95% CI [-43.29, 2.05]. These findings suggest a different effect over time for the rTMS group compared to the other groups.

Attentional control

Full sample. Without restricting the BDI-II scores, we explored if there was an effect of the full sample with a 4 (group: rTMS, ABM, combination, control) X 2 (time: pre-assessment, post-assessment) mixed ANOVA on attentional control. The ANOVA revealed a significant difference in time ($F(1,104) = 27.10$, $p < .000$, $\eta^2 = .087$), representing a practice effect over time. However, no significant differences between the groups ($F(3, 104) = .467$, $p = .706$, $\eta^2 = .008$), as well as no significant interaction effect for time and group ($F(3, 104) = 1.325$, $p = .270$, $\eta^2 = .014$) on attentional control were observed.

Effects on mood, depression and anxiety scores

<u>Mood manipulation check results:</u>

Restricted sample

To examine the effects of the mood manipulations using film clips, a repeated measures ANOVA was conducted on self-report mood as measured by Likert scales before and after watching the mood inducing

movies (T1 and T2 in figure 1c respectively) on the full sample. The results yielded a significant main effect of time (F(1,71) = 170.7, p <. 001, η^2 = .706), where mood levels dropped (indicating an increase in negative mood) after watching the negative mood inducing movie, similarly for all groups. Mood levels were elevated after watching the positive movie (F(1,70) = 170.4, p < .001, η^2 = .709; T6 and T7 in figure 1c respectively).

To test whether participants' mood ratings changed after the rTMS and/or ABM manipulations, a 4 (group: rTMS, ABM, combination, control) X 2 (time: pre-manipulation, post-manipulation, T3 and T4 in figure 1c respectively) mixed ANOVA on Likert scales was performed. The ANOVA revealed no significant effects of group (p = .88) or interaction effects (p = .97). There was a significant effect of time (F(1, 68) = 58.487, p < .001, η^2 = .462), where all groups reported an increase in negative mood, possibly due to fatigue.

To test whether groups responded differently in their overall mood rating after a stressful task, another 4 (group: rTMS, ABM, combination, control) X 2 (time: pre-stress task, post-stress task; T5 and T6 in figure 1c respectively) mixed ANOVA on Likert scales was performed. The ANOVA revealed no significant effects of group (p = .78) or interaction effects (p =.61). Again, there was a significant effect of time (F(1, 68) = 70.656, p <. 001, η^2 = .503), where all groups reported an expected decrease in mood.

Questionnaire results:

Full sample

The same analyses were exploratively repeated on the full sample, which yielded similar results as the for the restricted sample.

BDI. A 4 (group: rTMS, ABM, combination, control) X 3 (time: baseline, 3 day follow up, 3 week follow up) mixed ANOVA on BDI-II scores was performed, which revealed no significant main effect of time F(2,178) = 1.244, p = .291, η^2 = .002, nor for group F(3,89) = 1.34, p = .267, η^2 = .003, nor an interaction of both, F(6,178) = 0.47, p = .833, η^2 = .003.

STAI-T. A 4 (group: rTMS, ABM, combination, control) X 3 (time: baseline, 3 day follow up, 3 week follow up) mixed ANOVA on STAI-T scores was performed, which revealed no significant main effect of time F(2,186) = 2.244, p= .089, η^2 = .003, nor for group F(3,93) = 0.375, p = .771, η^2 = .001, nor an interaction of both, F(6,186) = 0.497, p =.810, η^2 = .002.

NA. A 4 (group: rTMS, ABM, combination, control) X 3 (time: baseline, 3 day follow up, 3 week follow up) mixed ANOVA on negative affect (NA) scores was performed, which revealed no significant main effect of time F(2,194) = 1.62, p =.2, η^2 = .003, nor for group F(3,97) = 0.02, p = .918, η^2 < .001, nor an interaction of both, F(6,194) = 0.89, p = .502, η^2 = .005.

PA. A 4 (group: rTMS, ABM, combination, control) X 3 (time: baseline, 3 day follow up, 3 week follow up) mixed ANOVA on positive affect (PA) scores was performed, which revealed no significant differences between the groups, F(3,97) = 1.3, p = .279, η^2 = .03. However, a significant main effect of time F(2,194) = 5.03, p = .007, η^2 = .001, as well as a small significant interaction between time and group was found, F(6,194) = 2.16, p = .048, η^2 = .001.

S-Figure 1

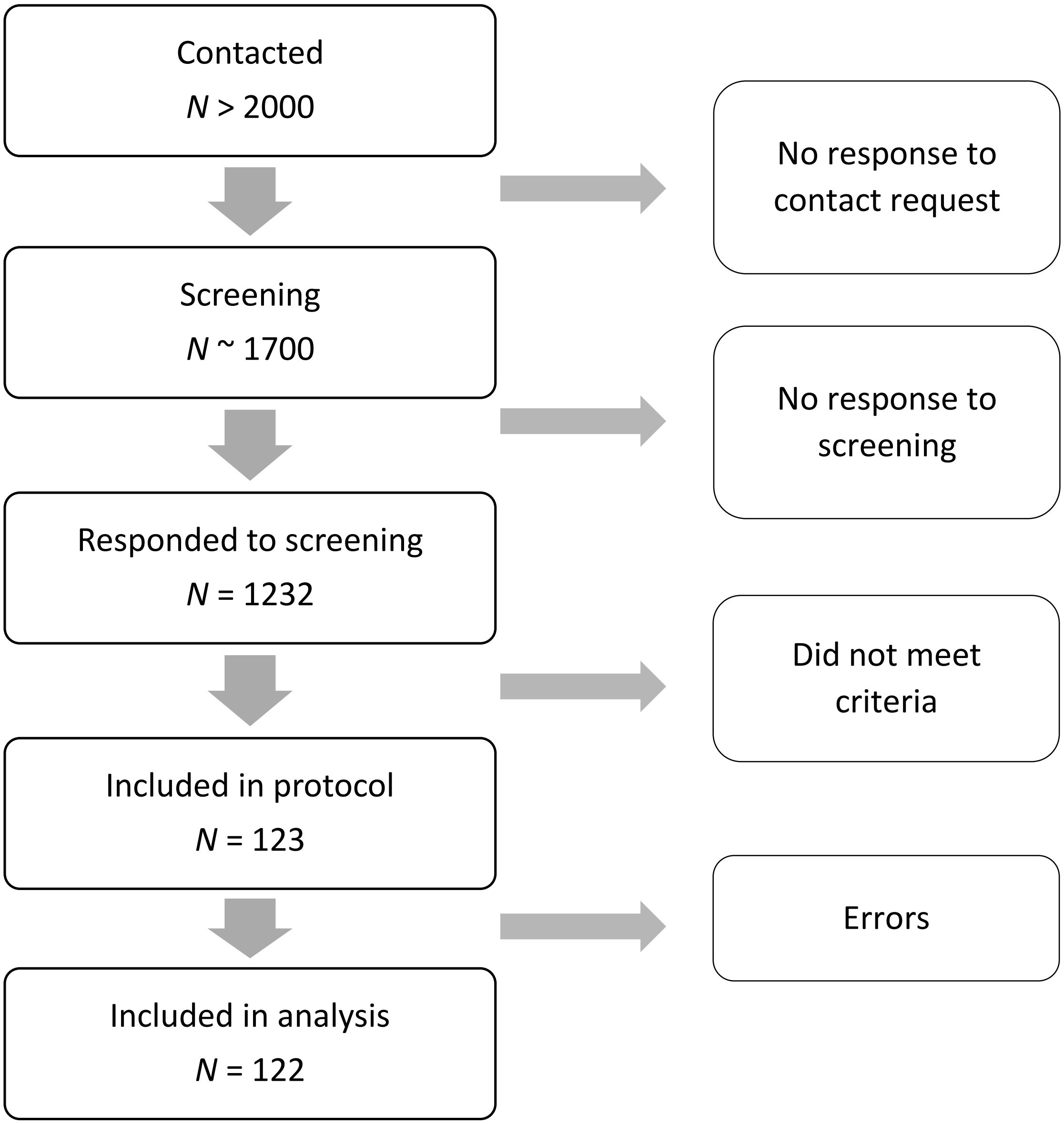

Flowchart subject inclusion. Of the ~2000 people who were contacted through email contact, around 1700 responded and were sent further information and screening questionnaires. 1232 participants responded and completed to the screening process. Out of these, only 123 Dutch or German-speaking participants fit all criteria were invited to the lab. In the final analyses, data of 122 subjects were used.

S-Table 1
Sample sizes per group and outcome measurement

**Sample sizes complete sample**	**rTMS**	**ABM**	**Combination**	**Control**	**Sample sizes after BDI-II restriction (≥9)**	**rTMS**	**ABM**	**Combination**	**Control**
Tasks									
ABM	32	30	30	30	ABM	21	18	18	15
AC	32	29	29	29	AC	20	18	19	15
ABM[1]	27	26	30	26	ABM[1]	19	15	18	13
AC[1]	28	27	27	26	AC[1]	17	16	19	14
Questionnaires									
Baseline[2]	32	30	30	30	Baseline[2]	20	18	20	15
BDI-II[3]	24	22	26	23	BDI-II[3]	16	15	15	10
STAI-T[3]	27	24	26	24	STAI-T[3]	18	15	15	11
NA[3]	27	24	26	24	NA[3]	18	15	15	11
PA[3]	27	24	26	24	PA[3]	18	15	15	11

[1]Sample size after removal of outliers and incomplete cases
[2]Sample size for BDI-II, STAI-II and PANAS questionnaires at baseline (during labsession)
[3]Sample size per questionnaire specifically at baseline (during labsession), 3 days later (online measurement) and 3 weeks later (online measurement), after removal of outliers and incomplete cases
Note: rTMS = repetitive transcranial magnetic stimulation; *ABM* = attentional bias modification; *AC* = attentional control*; BDI-II* = Becks depression inventory*; STAI-T* = Spielberger trait anxiety inventory*; PA* = positive affect from the Positive and Negative Affect Scale (PANAS)*; NA* = negative affect from the PANAS;

S-Table 2

Change of Bayes Factor (BF) size as a function of varying r scale fixed effect priors of 4 x 2 mixed ANOVA on attentional bias.

**Prior (r scale fixed effects)**	**Group BF_{10}**	**Time BF_{10}**	**Group * Time BF_{10}**
0.1	0.679	0.611	0.293
0.25	0.384	0.383	0.050
0.5	0.158	0.209	0.005
0.75	0.074	0.146	0.00076
1	0.039	0.109	0.00016

S-Table 3

Reaction times in milliseconds averaged per group per time point of measuring attention bias for positive and negative trials on the full sample. Median and standard deviation are reported.

	**rTMS (*N*=32)**	**ABM (*N* = 30)**	**Both (*N* = 30)**	**Control (*N* = 30)**
Positive trials pre	642.53 (68.39)	644.38 (56.74)	656.92 (48.59)	661.88 (72.21)
Negative trials pre	646.97 (75.31)	649.38 (54.65)	646.37 (57.79)	657.78 (64.10)
Bias pre	-4.44 (62.65)	-5 (52.66)	10.55 (24.07)	4.10 (45.41)
Positive trials mid	604.55 (52.90)	602.90 (57.59)	594.27 (56.25)	617.47 (68.0)
Negative trials mid	606.63 (70.54)	607.45 (60.87)	606.17 (68.27)	616.18 (53.96)
Bias mid	-2.08 (45.25)	-4.55 (35.45)	-11.90 (50.20)	1.28 (42.99)
Positive trials post	600.80 (59.12)	582.46 (66.59)	597.35 (80.18)	613.20 (60.94)
Negative trials post	603.09 (63.05)	608.23 (58.08)	603.48 (75.15)	622.67 (57.31)
Bias post	3.02 (32.28)	-17.13 (44.18)	-5.85 (33.40)	-0.63 (40.48)

Note: rTMS = repetitive transcranial magnetic stimulation; *ABM* = attentional bias modification

S-Table 4
Reaction times in milliseconds averaged per group per time point of measuring attention bias for positive and negative trials on ***restricted sample***. Median and standard deviation are reported.

	**rTMS (*N*=20)**	**ABM (*N* = 18)**	**Both (*N* = 19)**	**Control (*N* = 15)**
Positive trials pre	646.20 (69.22)	639.69 (53.71)	652.76 (55.62)	644.97 (60.389)
Negative trials pre	651.08 (81.50)	647.47 (54.45)	638.79 (63.56)	647.63 (70.97)
Bias pre	-4.88 (76.72)	-7.78 (57.66)	13.97 (23.54)	-2.67 (47.89)
Positive trials mid	610.90 (53.69)	600.56 (57.35)	593.76 (60.44)	612.10 (72.40)
Negative trials mid	608.73 (75.11)	602.92 (63.26)	608.32 (66.37)	625.57 (42.93)
Bias mid	2.18 (44.54)	-2.36 (35.04)	-15.55 (50.41)	-13.47 (47.42)
Positive trials post	596.68 (53.96)	571.92 (69.66)	599.13 (74.23)	609.67 (55.51)
Negative trials post	603.88 (64.07)	600.67 (54.47)	601.61 (76.34)	625.43 (57.58)
Bias post	3.50 (37.34)	-19.92 (32.33)	-1.05 (39.08)	0.50 (46.14)

Note: rTMS = repetitive transcranial magnetic stimulation; *ABM* = attentional bias modification

References:

1. Price, R. B., Kuckertz, J. M., Siegle, G. J., Ladouceur, C. D., Silk, J. S., Ryan, N. D., Dahl, R.E. & Amir, N. (2015). Empirical recommendations for improving the stability of the dot-probe task in clinical research. *Psychological assessment*, *27*(2), 365.
2. Waechter, S., Nelson, A. L., Wright, C., Hyatt, A., & Oakman, J. (2014). Measuring attentional bias to threat: Reliability of dot probe and eye movement indices. *Cognitive Therapy and Research*, *38*(3), 313-333.
